# Potential Genes Associated with COVID-19 and Comorbidity

**DOI:** 10.7150/ijms.67815

**Published:** 2022-01-24

**Authors:** Shanshan Feng, Fuqiang Song, Wenqiong Guo, Jishan Tan, Xianqin Zhang, Fengling Qiao, Jinlin Guo, Lin Zhang, Xu Jia

**Affiliations:** 1 College of Medical Technology, Chengdu University of Traditional Chinese Medicine, Chengdu, Sichuan, China.; 2 Non-coding RNA and Drug Discovery Key Laboratory of Sichuan Province, Chengdu Medical College, Chengdu, Sichuan, China.; 3 Department of medical Laboratory, The General Hospital of Western Theater Command, Chengdu, China.; 4 Chengdu Medical College, Chengdu, China.; 5 School of Basic Medical Sciences, Chengdu Medical College, Chengdu, Sichuan, China.; 6 Key Laboratory of Systematic Research of Distinctive Chinese Medicine Resources in Southwest China, Chengdu University of Traditional Chinese Medicine, Chengdu, China.; 7 Chongqing Key Laboratory of Sichuan-Chongqing Co-construction for Diagnosis and Treatment of Infectious Diseases Integrated Traditional Chinese and Western Medicine, Chengdu University of Traditional Chinese Medicine, Chengdu, China.; 8 Department of Pharmacy, Shaoxing People's Hospital, Shaoxing Hospital, Zhejiang University School of Medicine, Shaoxing, China.

**Keywords:** COVID-19, comorbidity, bioinformatics, susceptibility gene, disease severity, SARS-CoV-2

## Abstract

Hypertension, diabetes mellitus, and coronary artery disease are common comorbidities and dangerous factors for infection and serious COVID-19. Polymorphisms in genes associated with comorbidities may help observe susceptibility and disease severity variation. However, specific genetic factors and the extent to which they can explain variation in susceptibility of severity are unclear. Therefore, we evaluated candidate genes associated with COVID-19 and hypertension, diabetes mellitus, and coronary artery disease. In particular, we performed searches against OMIM, NCBI, and other databases, protein-protein interaction network construction, and GO and KEGG pathway enrichment analyses. Results showed that the associated overlapping genes were* TLR4*, *NLRP3*, *MBL2*, *IL6*, *IL1RN*, *IL1B*,* CX3CR1*, *CCR5*, *AGT*, *ACE*, and* F2*. GO and KEGG analyses yielded 302 GO terms (q < 0.05) and 29 signaling pathways (q < 0.05), respectively, mainly including coronavirus disease-COVID-19 and cytokine-cytokine receptor interaction. *IL6* and *AGT* were central in the PPI, with 8 and 5 connections, respectively. In this study, we identified 11 genes associated with both COVID-19 and three comorbidities that may contribute to infection and disease severity. The key genes IL6 and AGT are involved in regulating immune response, cytokine activity, and viral infection. Therefore, RAAS inhibitors, AGT antisense nucleotides, cytokine inhibitors, vitamin D, fenofibrate, and vaccines regulating non-immune and immune factors could be potential strategies to prevent and cure COVID-19. The study provides a basis for further investigation of genes and pathways with predictive value for the risk of infection and prognosis and could help guide drug and vaccine development to improve treatment efficacy and the development of personalised treatments, especially for COVID-19 individuals with common comorbidities.

## Introduction

SARS-CoV-2, with its highly infectious property, has spread quickly worldwide, leading the WHO to officially and publicly declare COVID-19 a global pandemic 1. Up to 6 October 2021, WHO reported 235,673,032 cases worldwide, including 4,814,651 deaths 2. SARS-CoV-2 can degrade angiotensin-converting enzyme 2 (ACE2) on the cell membrane. Virions bind to exfoliated ACE2, resulting in a decrease of free ACE2, disrupting the balance of human ACE/ACE2, aggravating inflammatory reactions, and promoting interstitial and endothelial fibrosis as well as myocardial hypertrophy 3, 4. The virus also causes cytokine storms, alters cytoskeleton dynamics, strengthens vasoconstriction, and causes serious complications such as acute respiratory distress syndrome (ARDS) and pulmonary fibrosis 5, 6, which seriously harm human health. At present, several vaccines for SARS-CoV-2 have been put on the market. However, the virus is prone to mutation and its genome is characterised by high diversity and frequent recombination, leading to weak protective power and insufficient duration of protection 7. Additionally, frequent cross-species transmission further emphasises the importance of fundamental preventive and therapeutic strategies.

The envelope of SARS-CoV-2 mainly comprises four constitutive proteins, the spike (S), membrane (M), envelope (E), and nucleocapsid (N) proteins. This viral S protein can bind to the host ACE2 and split into S1 and S2 subunits by transmembrane serine protease 2 (TMPRSS2). Human ACE2 can interact with the receptor-binding domain (RBD) on the S1 subunit, and the fusion peptide on the S2 subunit enters the infected host cell membrane 8, 9. The RBD region of the S1 subunit can be used as a vital target for developing drugs and vaccines 10. The S1/S2 subunit has a recognition site for furin, and S protein cleavage and pre-activation by furin increase viral invasiveness 11. Compared to the S protein of SARS-CoV, the affinity of SARS-CoV-2 with human ACE2 increased at least ten times with stronger infectivity 12. The E protein is the most highly conserved in SARS-CoV-2. It mainly participates in the formation of the viral envelope, nucleocapsid assembly, pore protein formation, selective transport of Ca^2+^ via pore proteins, as well as virus particle assembly and release from host cells 13. Recombinant SARS-CoV-2 lacking E protein shows decreased viral titres and impaired viral maturation and reproduction 14, suggesting that E protein is a therapeutic target for latent infection. The M protein in SARS-CoV-2 contains three transmembrane domains and one conserved domain. It interacts with S and E proteins in the viral envelope, participating in the assembly and release of virus particles, enabling the host to produce neutral antibodies. It is expected to become a potent immunogen for treating COVID-19 9, 15. N protein, situated at the core of the virion, is a highly conserved phosphorylated protein involved in the replication and translation of viral RNA and formation and maintenance of the ribonucleoprotein complex, pivotal for viral protein assembly 16. It is produced in large quantities during infection and has high immunogenicity, which is important for vaccine development 17. The S protein binds to ACE2 and immune receptors, such as Toll-like receptors (TLRs) and NOD-like receptors (NLRs) 18. A few studies characterised the binding relationship between host cell receptors and structural proteins other than S protein. Accordingly, it is necessary to study host-virus interactions to clarify the pathogenic mechanism for finding new ways of preventing and curing COVID-19.

During the course of the COVID-19 epidemic, differences in rates of infection and disease outcomes among populations have been observed. There are various risk factors for COVID-19, among which human genetic polymorphisms play an important role. Eduardo et al. 19 showed that the prevalence rate in men was significantly higher than that in women in most age groups in Chile. The important receptor ACE2 for SARS-CoV-2 binding is on the X chromosome. Because males are hemizygous, they have a higher risk of overexpressing ACE2 mutations, resulting in increased susceptibility or disease severity 20. The rate of SARS-CoV-2 invasion in humans with A blood group is also significantly higher than that in the O blood group 21-23. Two genome-wide association studies (GWAS) have shown that 3P21.31 and the 9q34 region containing ABO blood group sites are significantly associated with severe COVID-19 24, 25. The IFNL4 variant rs12979860 is associated with the presence of COVID-19 in the Spanish population, independent of gender, age, and disease severity, which may be related to reduced virus clearance 26. The status of the *PNPLA3* variant rs738409 and the *TLL-1* variant rs17047200 is related to SARS-COV-2-induced infection 27. The frequencies of *TMPRSS2* variants rs12329760 and rs2298659 are associated with the severity of COVID-19, especially in young men and older women. It has also been predicted that rs12329760 could affect the stability of TMPRSS2 protease and have a protective effect on patients 28. The frequency of rs12329760 is higher in East Asia than in European countries, and the elevated frequency is associated with a lower fatality rate 29. In Madrid, a sequencing analysis of 23 cases of familial multiple sclerosis has shown that *TMPRSS2* synonymous variants rs61735792 and rs61735794 were significantly associated with infection, whereas ACE2 and furin were not significantly associated with SARS-COV-2 infection 30. However, previous studies have reported that the *ACE2* variant rs41303171 is significantly more frequent in patients with COVID-19 than in controls 31. Furthermore, another study of an Italian population has shown that the furin encoded by the *PCSK3* variant rs769208985 is associated with COVID-19 infection 32. These results suggest that different populations may have different alleles and genotypes that contribute to COVID-19 infection and severity.

Most patients with COVID-19 have comorbidities, which may be risk factors for susceptibility and severity 33, 34. In particular, hypertensive disease, diabetes mellitus (DM), and coronary artery disease (CAD) are frequent COVID-19 comorbidities. Guan et al. 35 reported that 399 (25.1%) of the 1590 COVID-19 patients in China had comorbidities. Hypertension, DM, and CAD accounted for 16.9%, 8.2%, 3.7%, and 1.3% of these comorbidities, respectively, and 130 patients had at least two comorbidities. After adjusting for age and smoking status, DM and hypertension were compound endpoint risk factors for entering the intensive care unit, intrusive ventilation, and even death. A cohort study including 201 COVID-19 cases in Wuhan showed that hypertensive disease, DM, and CAD were the more frequent complications, accounting for 19.4%, 10.9%, and 4%, respectively. Comorbidities have been observed in 46.3% and 67.1% of critical and severe cases, respectively, compared with 37.8% of moderate COVID-19 cases 36. Several meta-analyses have also confirmed that hypertensive disease, DM, and CAD were more frequent comorbidities of COVID-19. These comorbidities are more frequent in severe patients and are risk factors for disease progression 37-40. However, the role and molecular mechanism underlying the association between common comorbidities and COVID-19 remain unclear. The polymorphism and mutation expression patterns of genes related to these diseases may lead to susceptibility to SARS-CoV-2 and different outcomes 41. Further research is needed to be conducive to preventing and curing COVID-19.

Variants with increased transmissibility, including delta (B.1.617.2), lambda (C.37), gamma (P1), beta (B.1.351), and alpha (B.1.1.7), have recently appeared and spread worldwide 42-44. The spread of COVID-19 caused by the delta variant was more rapid and severe than other genotypes 45, while viral mutations render established vaccines less protective, causing breakthrough infection 46. The mutant strains can impact response measures, requiring preventive intervention even after vaccination. Identifying factors associated with susceptibility and severity and elucidating their biological mechanisms are crucial for preventing and treating COVID-19. In addition to meta-analyses and retrospective clinical studies showing that hypertension, DM, and CAD are common comorbidities and potential risk factors for infection and acute COVID-19, further evidence showed that DM and hypertension are risk factors for CAD, which can cause the three comorbidities to co-occur 47, placing patients at a greater risk of developing COVID-19 and suffering from worse clinical symptoms. However, studies of the mechanism underlying the association between the three comorbidities and COVID-19 are lacking. Identifying genes associated with comorbidities is a quintessential research goal to guide the detection of high-risk groups, prevent the disease, and treat COVID-19 patients with common comorbidities.

Bioinformatics approaches can be used to integrate and analyse genetic information and other data types, explore the mechanisms underlying specific pathological conditions, and identify new therapeutic targets. NCBI, OMIM, and other common databases were used to identify genes associated with COVID-19 as well as hypertension, DM, and CAD to predict susceptibility and severity in this study. A protein-protein interaction (PPI) network was generated using Cytoscape, and Gene Ontology (GO) and Kyoto Encyclopedia of Genes and Genomes (KEGG) pathway enrichment analyses were performed. Nonetheless, the candidate susceptibility genes require further validation in larger studies to identify potential therapeutic targets for new adjuvant drugs and vaccines and develop personalised preventive measures and treatment plans.

## Materials and methods

### Collection of diseases-related genes

Disease-related genes were identified using the search terms “hypertension”, “diabetes mellitus”, “coronary artery disease”, and “COVID-19.” Four databases were used to screen for genes related to hypertension, DM, CAD, and COVID-19, including OMIM (http://www.omim.org/), KMDB/MutationView (http://mutationview.jp/MutationViewV2Server/), DisGeNET database (https://www.disgenet.org/), and NCBI database 48. OMIM is an online catalogue of human genes and their related mutations based on human Mendelian genetics and diseases. KMDB/*MutationView* provides human disease-related gene mutations covering the eye, ear, heart, tumor, autoimmune disease, muscle, and blood disease genes. The DisGeNET database collects information for associations between mutation sites and diseases. The NCBI database includes information for COVID-19-related genes.

### Identification of overlapping genes related to COVID-19 and comorbidities

The related genes between COVID-19 and three comorbidities were cross-analysed using Bioinformatics (http://www.bioinformatics.com.cn/). An online platform 49 was used to draw Venn diagrams of common genes associated with the diseases.

### Functional enrichment analysis and network visualization of intersecting genes

R (version 4.1.0) was used to load the Bioconductor (https://www.bioconductor.org/) packages “stringi,” “DOSE,” “apeglm,” “BiocManager,” “clusterProfiler,” and “pathview” to carry out GO analysis for common disease genes, KEGG analysis, and visual graphic display. The screening criteria for enrichment were p < 0.01 and q < 0.01. The p-values were estimated using the hypergeometric test and corrected for multiple testing with the Benjamini-Hochberg method. Additionally, q-values were estimated to control for the false discovery rate. The R script is provided in Supplementary File S1. A network graph of intersecting genes was constructed using Cytoscape (version 3.7.0).

### Construction of a PPI network and determination of core targets

The STRING database was used to visualise the PPI network, which reveals the strength of relationships and connections between proteins corresponding to intersecting genes. Each node in the PPI represents a protein, and the connections between nodes illustrate interactions. The list “Multiple proteins” and the organism “*Homo sapiens*” were selected as the screening conditions. In basic settings, high confidence of 0.700 was chosen 50. Cytoscape was used to build and visualise PPI networks. The topological parameters were analysed using Cytoscape, and the key genes were searched using cytoHubba. Genes with a degree value greater than the mean were defined as key genes.

## Results

### Disease-related mutations and intersecting genes

**Figure 1:**
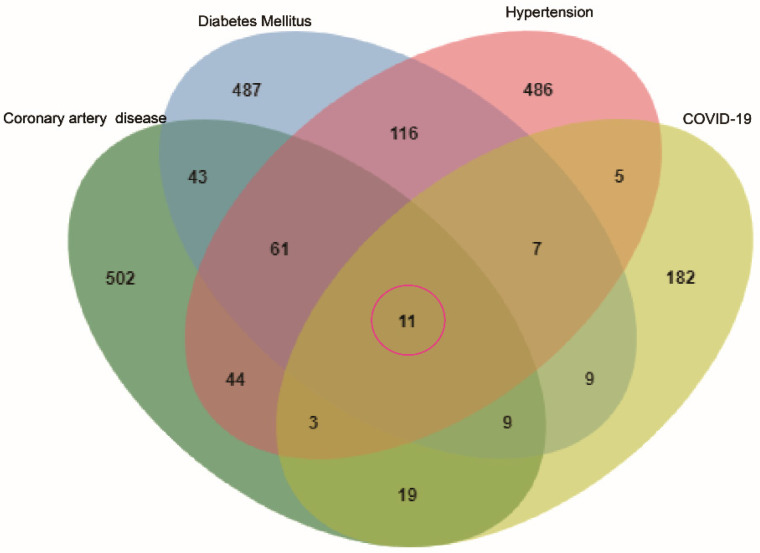
** Venn diagram of the intersection genes to the three comorbidities of COVID-19.** Notes: The 11 overlapping genes of comorbidities of hypertension, DM, CAD and COVID-19 were *TLR4, NLRP3, MBL2, IL6, IL1RN, IL1B, CX3CR1, CCR5, AGT, ACE, F2*.

To identify genes associated with hypertension, DM, CAD, three databases (DisGeNET, OMIM, and KMDB) were used. After removing duplicate data, 733, 743, and 692 genes associated with hypertension, DM and CAD were identified, respectively. After superposition and the deletion of duplicate data for 150 genes related to COVID-19 from the SARS-CoV-2 data in NCBI and 108 genes from the gene map of OMIM, 245 genes were obtained. Then, a Venn diagram of overlapping genes was drawn using an online Bioinformatics platform. Eleven common genes (*TLR4*, *NLRP3*, *MBL2*, *IL6*, *IL1RN*, *IL1B*,* CX3CR1*, *CCR5*, *AGT*, *ACE*, and *F2*) were identified (Figure 1). Polymorphisms of these genes may be predictors of susceptibility and severity of COVID-19. Disease-related mutations genes are listed in Supplementary Table S1.

### Enrichment analysis of intersecting genes

#### GO analysis of intersecting genes

**Figure 2:**
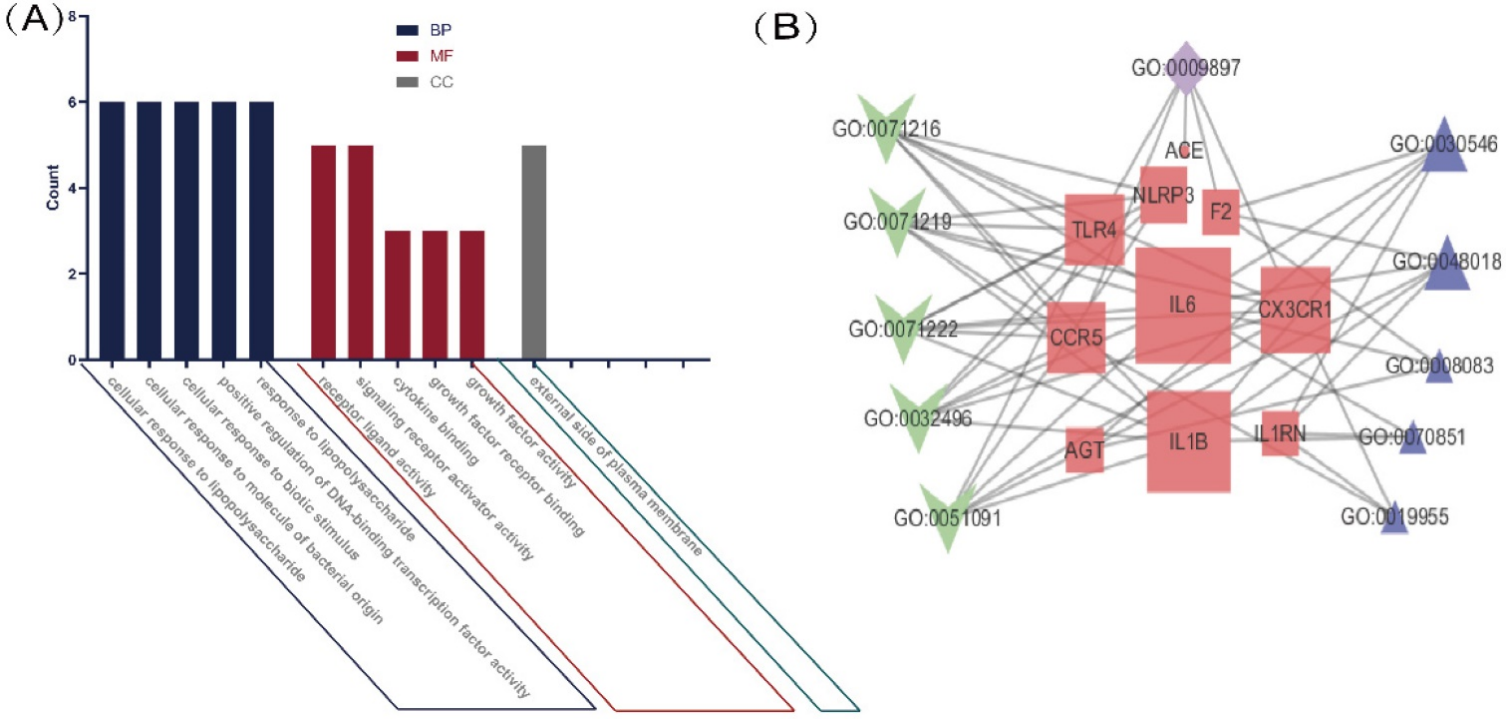
** GO analysis of intersection genes and network visualization.** Notes: **(A)** GO analysis of intersection genes. The value on the Y-axis represents counting the intersection genes in each GO term. Blue represents BP, red represents MF, and green represents CC. The X-axis is the GO term, respectively the top 5 BP, MF and the only cell component enriched according to the count value. **(B)** Network visualization of gene and GO term. Red represents the common gene, green represents BP, purple represents CC, blue represents MF, the larger the shape means the more connections in the whole network.

We performed a GO enrichment analysis of 11 genes associated with COVID-19 and common comorbidities. The results showed that genes were enriched in 286 biological processes (BP), including lipopolysaccharide, bacterial molecules, biological stimulation, acute inflammation, regulation of transcription factors, cytokine activity, JAK-STAT receptor signaling pathway, immunity, and metabolic regulation. The lateral plasma membrane was the only enriched cellular component (CC). The genes were associated with 15 molecular functions (MF), including cytokine binding and activity, chemokine binding and receptor activity, and cell receptor-ligand binding. The top five BP, MF, and CC terms are shown in Figure 2A, and a network map of common intersecting gene-related GO terms was constructed using Cytoscape (Figure 2B). In Table 1, detailed results of the GO enrichment analysis are provided. The results suggested that three comorbidities of COVID-19 were mainly related to metabolic and immune system disorders, insufficient or excessive response to external stimuli, and changes in the expression and activity of cytokines, resulting in changes in susceptibility and severity.

#### KEGG pathway analysis of intersection genes

**Figure 3:**
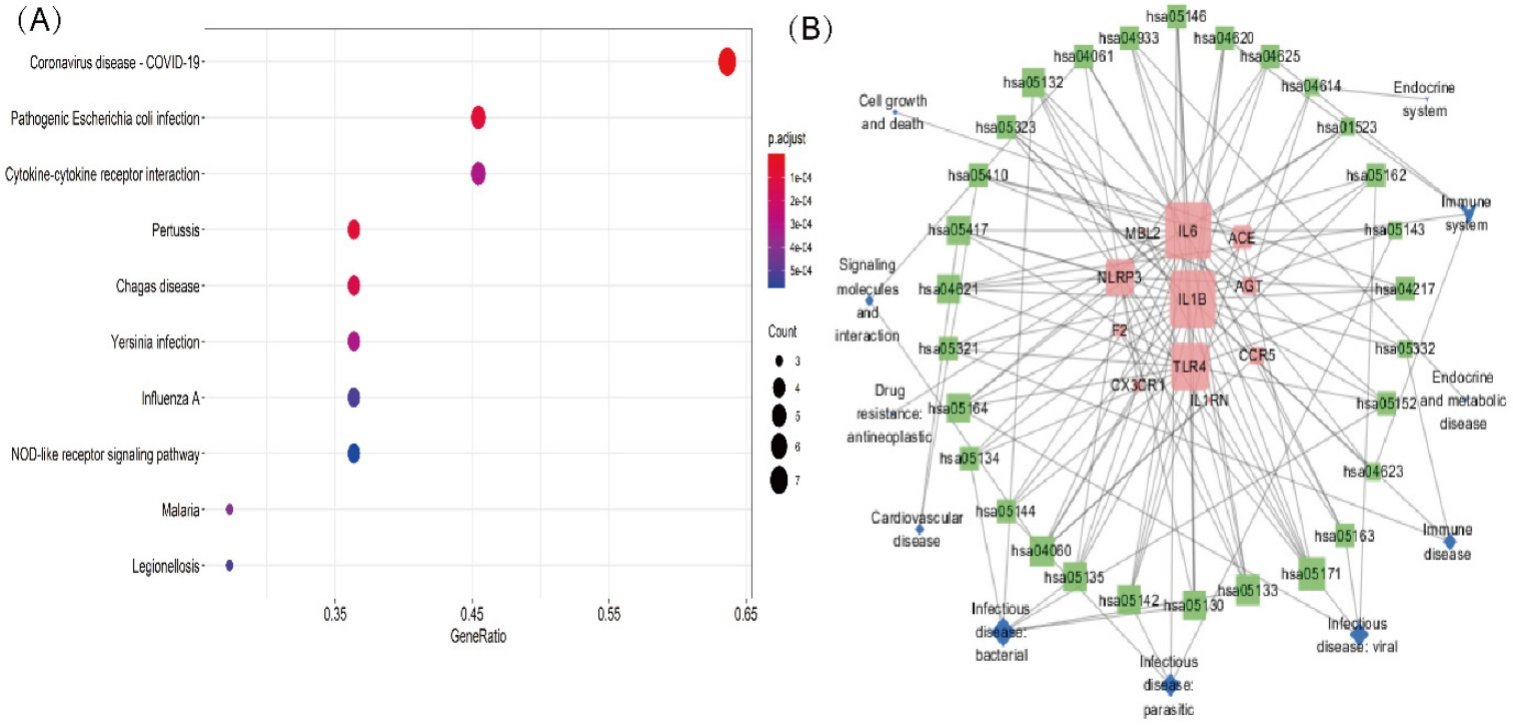
** KEGG analysis of intersection genes and network visualization.** Notes: **(A)** KEGG analysis of intersection genes. A higher GeneRatio value indicates a higher enrichment level; The bigger circle indicates more target genes in the pathway. The color ranges from blue to red of circles indicating that the adjusted p-values increases from small to large and the enrichment results are more significant. **(B)** Network visualization of intersecting genes and enriched KEGG pathways. Red represents the common gene, green represents the KEGG pathway and blue represents the upper-level classification of the KEGG pathway. The larger the shape, the more connections in the entire network.

**Figure 4:**
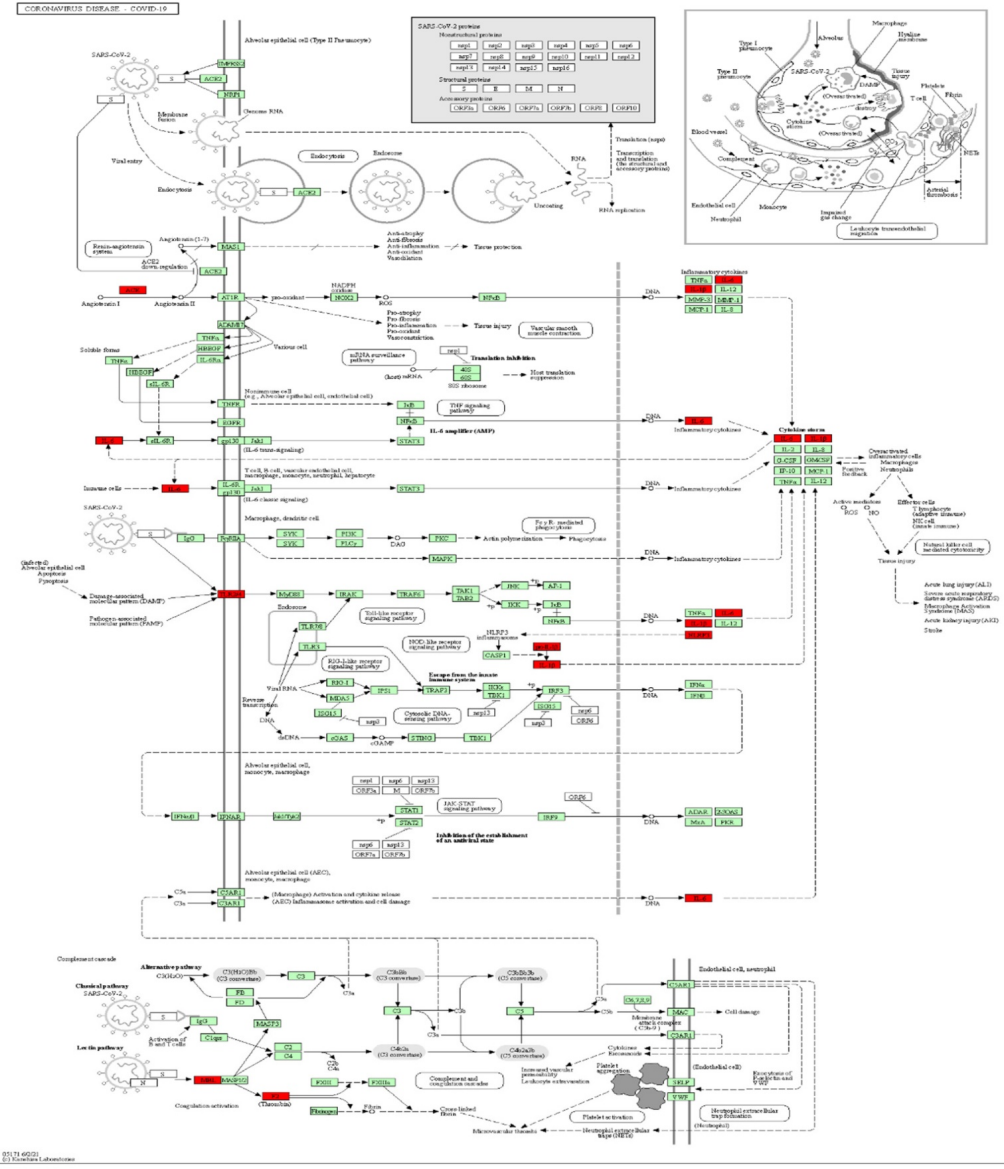
** Mapping of intersection genes in the Coronavirus disease-COVID-19 pathway.** Notes: The genes in red are mappings of intersection genes in Coronavirus disease-COVID-19 pathway. The intersection genes were mainly mapped in cytokines involved in the inflammatory process. *F2* and *TLR4* mediate coagulation process and immune recognition against the viral infection process, respectively.

A KEGG pathway analysis was performed to identify pathways involving genes associated with COVD-19 and common comorbidities. In total, 29 KEGG pathways were enriched, including pathways related to human diseases, biological systems, environmental information processing, cellular processes, including viruses, bacteria, parasitic infectious diseases, endocrine metabolic diseases, cardiovascular diseases, immune diseases, signaling molecules and interactions, cell growth and death, immune system, and endocrine system. The top ten pathways were coronavirus disease-COVID-19, cytokine-cytokine receptor interaction, pertussis, Chagas disease, *Yersinia* infection, influenza A, lipid and atherosclerosis, *Salmonella* and *Escherichia coli* infection, and NLR pathway (Figure 3A). A network diagram of the intersection gene-KEGG pathways was constructed using Cytoscape (Figure 3B). The common genes were most highly enriched in coronavirus disease-COVID-19, with the highest GeneRatio value. A map of the overlapping genes in coronavirus disease-COVID-19 pathway was obtained by KEGG mapping online tool (Figure 4). These common genes were mainly involved in the inflammatory process caused by COVID-19-related cytokines, such as IL6 and IL1β. F2 was involved in the coagulation process and TLR4-mediated immune recognition against virus infection. In addition, interactions between viral proteins and cytokines, inflammatory diseases, diabetic complications, hypertrophic cardiomyopathy, the renin-angiotensin system, and other pathways were also identified in the enrichment analysis. The results, summarised in Table 2, suggested that the genes were involved in infectious and metabolic diseases, mainly by affecting the interactions between viral proteins and cytokine receptors, regulating immune and inflammatory responses. Detailed KEGG and GO analysis results are provided in Supplementary Table S2.

### PPI network analysis

**Figure 5:**
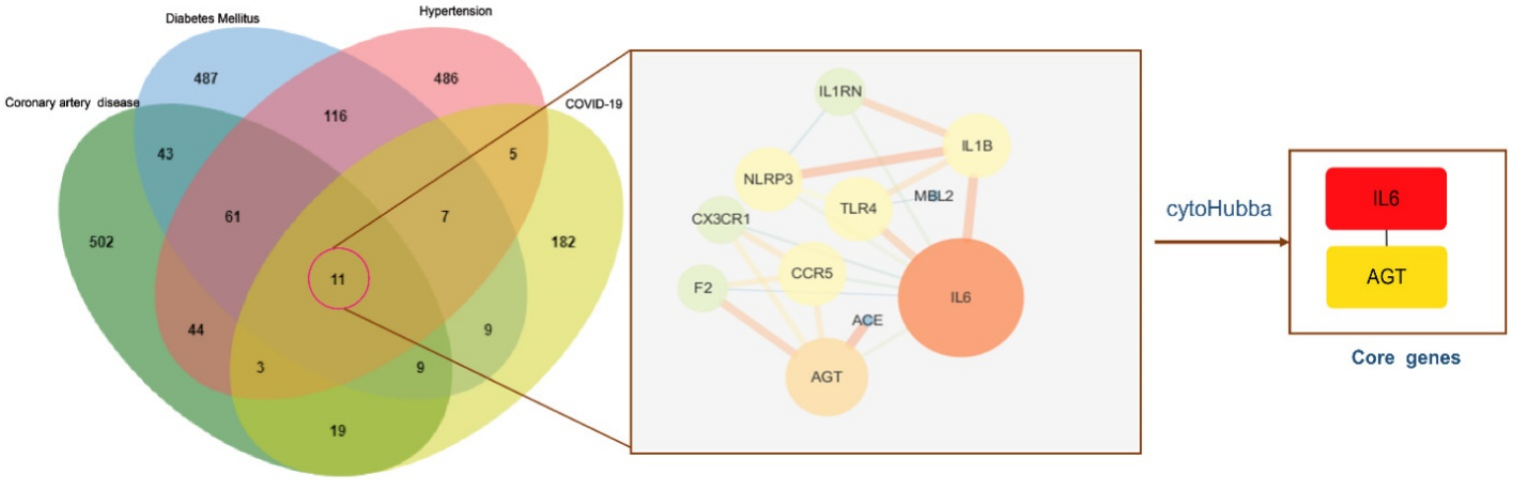
** PPI networks of intersection genes and core genes.** Notes: The bigger circle represents greater degree; Lines between nodes represent interactions between proteins encoded by genes, with thicker lines indicating stronger interactions; *IL6* and *AGT* were selected as the core genes, and the degree value was 8, 5, respectively. *IL6* showed the highest number of connections.

To understand the interactions between genes and identify key genes among the 11 common genes associated with COVID-19 and comorbidities, the STRING database was used, and the PPI network was presented using Cytoscape software (Supplementary Table S3). Lines between nodes represent interactions between proteins, where thicker lines indicate stronger interactions. *IL6* and *AGT* were selected as core genes, with degree values (i.e., the number of connections) of 8 and 5, respectively (Figure 5), indicating that they were central in the PPI and may be more closely related to the disease state. *IL6* showed the highest number of connections and was most closely related to other common genes. It was implied that *IL6* may have a vital effect on the infection and pathogenesis and can be used as a key starting point for prediction and treatment.

## Discussion

We constructed a gene-protein interaction network based on common genes associated with three common comorbidities and COVID-19. Eleven common genes were found, including* TLR4*, *NLRP3*, *MBL2*, *IL6*, *IL1RN*, *IL1B*,* CX3CR1*, *CCR5*, *AGT*, *ACE*, and* F2*. These genes may be potential risk predictors of COVID-19. GO and KEGG enrichment analyses further indicated that these 11 common genes were related to infection, inflammation, cytokine activity, and receptor-binding. Our results were similar to those of another bioinformatics study. The hub genes related to COVID-19 were enriched in the immune and inflammatory reactions, and cytokine-cytokine receptor interaction pathway 51, suggesting the key mechanisms and pathways underlying SARS-CoV-2 infection.

Previous work has demonstrated that the core gene *IL6* is connected to a higher risk of SARS-CoV-2 infecting and pathopoiesia 52. IL6 responds to tissue damage and infection and is secreted by diverse cells as a proinflammatory cytokine, such as fibroblasts, keratinocytes, and macrophages 53. Higher IL6 levels are commonly found in the three common comorbidities, which may lead to severe COVID-19. IL6 plays a key role in exacerbating inflammatory responses after infecting SARS-CoV-2. Excessive IL6 can lead to excessive activation of Th17 cells by activating the JAK-STAT pathway. Th17 cells produce numerous effectors (IL-17, IL-1β, IL-21, IL-22) that recruit neutrophils, upregulate fibrinogen, damage tissue, and cause systemic inflammatory symptoms 5, 54-56. A meta-analysis of COVID-19 and *IL6* polymorphisms has shown that carriers of the *IL6-*174C allele show more severe pneumonia 57. Another study has shown that mortality and prevalence are negatively correlated with the *IL6* rs1800795G allele in COVID-19 58. Immune system dysfunction and IL6 overexpression are characteristics of COVID-19, and elevated IL6 levels are related to respiratory failure and death 59. Recent research has shown that the common Asian haplotype C-T-T, represented by alleles rs1800796, rs1524107, and rs2066992, reduces the risk of severe COVID-19 via reduced IL6 expression 56. Clinical data has shown that the majority of patients show improved hypoxaemia and CT imaging and no significant adverse reactions after receiving tocilizumab, a monoclonal antibody binding with IL6 receptor, thus inhibiting the binding of IL6 to the receptor, to avoid damaging targeted cells and reducing inflammation 60. Considering the importance of IL6 in COVID-19-related pathomechanism, anti-IL6 therapies may be effective for severe COVID-19, including selective inhibitors of ADAM-17 or sgp130Fc, which inhibits IL6 signal transduction 61.

The core gene *AGT* encodes the precursor of angiotensin, which is highly produced in the liver and is the only substrate for renin. Angiotensin is converted to the active substance angiotensin (ANG) I/II in the renin-angiotensin system (RAS). It exerts physiological effects on target organs, including the kidney, blood vessels, and adrenal gland, and regulates the vascular tone, heart functions, and vascular remodelling 62. Sequence variation in this gene is associated with the risks of hypertensive disease, heart failure, and cardiovascular disease 63, 64. AGT is critical for ANG I /ANG II to maintain balance. The SARS-CoV-2-infected receptor ACE2 is an important member of the RAS system. Any changes in expression and function of the RAS element caused by genetic variation can lead to differences in susceptibility and severity to COVID-19 65, highlighting the importance of AGT in COVID-19. Cafiero et al. 66 studied the genotype and allele frequency distributions of *AGT* rs699 and found that asymptomatic patients have a lower frequency of the T/C genotype compared to symptomatic patients with COVID-19. The* AGT* rs699 SNP is potentially valuable tool for predicting clinical COVID-19 outcomes. The transfusion of *AGT* antisense nucleotides to inhibit AGT expression in male C57BL/6J mice for 14 days significantly reduced the expression of *TMPRSS2* mRNA in the lungs 67. It was suggested that suppression of *AGT* may have a protective effect against viral entry.

A relationship between *ACE* polymorphisms and COVID-19 has also been reported. *ACE* encodes an enzyme involved in regulating blood pressure and electrolyte balance, which catalyses transforming ANG I into ANG II with biologically active and participates in the imbalance of ACE2/ACE in the pathogenic process of SARS-CoV-2. The intron 16 of *ACE* is inserted/deleted 287bp to cause polymorphisms, which is currently the most studied polymorphism in *ACE*. There is a clear relationship between the polymorphism and blood ACE level 68. The I/D genotype is close to pathogenesis and progress of diabetic complications 69. The frequency and mortality of COVID-19 patients are closely connected to the *ACE*-I/D genotype that may be detected for predicting the ponderance of COVID-19 70. ACE2 is the vital receptor of SARS-CoV-2 binding host cell to infect humans. The expression and function of ACE are affected by its polymorphism, which plays a vital role in regulating ACE2 expression. The deficiency or overexpression of ACE alters ANG I accumulation, which may promote virus infection 68 and may explain why *ACE* is a potential susceptibility gene for COVID-19.

The intersecting gene *F2* encodes prothrombin. The rs1799963 and rs3136516 *F2* polymorphisms are associated with increases in the prothrombin level/activity and the risk of thrombosis 71, 72. Marker genes for arterial/venous thromboembolism include F2, and SNP genotyping of F2 will help identify the population most at risk of COVID-19-related thrombotic complications 73. Patients with coagulation abnormalities and severe infection-related systemic coagulopathies, including DIC or thrombotic microangiopathy, in severe COVID-19 show higher death rates. An elevated D-dimer level can increase the risk of death in severe cases; accordingly, anticoagulant treatment may reduce the mortality of COVID-19, as supported by studies of low-molecular-weight heparin revealing an improved prognosis 74. A study in Hong Kong has revealed that anticoagulants and antiplatelet drugs are related to lower hazard of critical COVID-19 75. As the correlation of COVID-19 coagulation abnormalities becomes clearer, the polymorphism of coagulation-related genes may be a considerable reason for the adverse progression of COVID-19 patients. Further studies of prothrombin *F2* polymorphisms in COVID-19 are needed.

Genes involved in immune system regulation may be related to variation in COVID-19 outcomes. Pathogens proliferate in the host in chronic illnesses, such as DM, affecting innate immunity 76. *MBL2* encodes a vital molecule in the innate immune system, a soluble mannose-binding lectin. An *MBL2* deficiency is related to the susceptibility of infectious diseases 77. The MBL2 variant rs5030737 is positively correlated with the number of COVID-19 cases but not with the number of deaths. The MBL2 variant rs1800450 is a positive correlation with the numbers of cases and deaths. The MBL2 variant rs1800451 is a negative correlation with the number of cases. These findings suggested that MBL2 inactivation is a risk factor for SARS-COV-2 infection 78, 79. Chemokine receptor-ligand interactions mediate inflammatory cell transport and pathogen-related immune responses. Early studies of *CCR5*-deficient mice have shown that the Th1 immune response is suppressed, increasing susceptibility to viral and bacterial infection 80. The deletion of the* CCR5* gene is related to a higher incidence of HCV 81. The *CCR5* Δ32 variant produces a truncated protein and observably decreases receptor formulation, suppressing the immune response 82, which may lead to increased virus susceptibility. A bioinformatics study has analysed the summary statistics of the GWAS dataset from patients with respiratory failure of COVID-19 and found that the first 10 BP involved the CCR5 83. In an epidemiological study, CCR5 Δ32 allele frequency positively correlated with COVID-19 mortality 84, suggesting CCR5 plays an important role in susceptibility and severity of COVID-19. Studies have shown that the number of non-classical monocytes is lower in CAD+SARS-COV-2 than in CAD-SARS-COV-2, and CX3CR1 expression on the surface was impaired. CX3CR1 mediated non-classical monocyte migration along with endothelial cells in the vascular system for antiviral immune response. Non-classical monocyte numbers and phenotypes were predictors of adverse clinical outcomes in CAD+SARS-COV-2-infected patients 85, suggesting that studies combined COVID-19 and comorbidities analysis can help detect different markers and improve therapeutic decision-making for different COVID-19 populations to efficient, personalised treatment.

Among KEGG signalling pathways identified in the study, TLR, NLR, and CLR signalling pathways play vital roles in initiating innate immune response, which can activate immune system and destroy immune tolerance state, which can disrupt immune tolerance state. It has been noted that cells expressing little or no ACE2 are still susceptible to the virus, suggesting that the S protein invades the host via receptors other than ACE2 86. Evidence from *in vitro* studies and computer simulations supports the interaction between S proteins and immune receptors, including NLRs, CLRs, and TLRs 87, consistent with the results of molecular docking study demonstrating binding between the S protein and TLRs, including TLR1, TLR4, and TLR6, in which the docking result showed that TLR4 had the highest binding energy 88. Activating TLR4 can increase ACE2 expression, promoting viral entry to cause excessive inflammation 89. The TLR4 pathway can produce IL6, a critical cytokine associated with cytokine storm, suggesting that TLR4 participates in SARS-CoV-2 invasion. NLRP3 inflammatory bodies composed of apoptosis-associated speck-like protein, caspase-1, and oligomeric NLRP3 contribute to severe COVID-19, and inflammatory bodies might be related to severe cytokine storms, resulting in ARDS and even death 90, 91. Anti-inflammatory and NLRP3 inflammatory body inhibitors could be potential therapeutic drugs for COVID-19.

The immune system is out of control to excessively release inflammatory mediators, resulting in a cytokine storm, a critical feature of COVID-19 recognized as the main reason causing serious outcomes. High levels of proinflammatory cytokines were observed in patients with COVID-19, such as IL1, IL1β, IL2, IL6, and TNF-α 92, 93. COVID-19 comorbidities, including hypertensive disease, DM, and CAD, are characterised by chronic inflammation 94. Most of the intersecting genes were associated with both COVID-19 and these comorbidities, playing a crucial role in inflammation. Mutations in genes encoding cytokines, such as *IL1B, IL1R1,* and* IL1RN*, likely cause a cytokine storm and critical COVID-19 95. Supportive and symptomatic treatment is necessary to control cytokine storms by inhibiting inflammation 96. Drugs targeting IL1, IL6, IL18, and interferon-γ, are effective in treating various cytokine storm-related syndromes 97. Therefore, this is an important area of research aimed at COVID-19 treatment.

Non-immune and immune factors are targets for the identification of new auxiliary drugs for COVID-19. The core genes *ATG* and* ACE* of the RAS are related to the ACE/ACE2 balance. The renin-angiotensin-aldosterone system (RAAS) blocker ACE-I/ARB has a protective effect against COVID-19, and RAAS inhibitors may be associated with a lower probability of death 98, 99. AGT antisense oligodeoxynucleotides inhibit AGT, providing another potential treatment. Interventions targeting the immune pathway are effective for preventing and curing COVID-19. A study has shown that the Lianhua Qingwen capsule can treat or interfere with the pathological process of COVID-19 via the NLR and TLR signalling pathways, NLRP33, and IL6 100. Additionally, metformin has anti-inflammatory activity. It can target mitochondrial electron transport and reduce IL6 levels by blocking ROS/CRAC, providing new curative pathways to weaken cytokine activity and thrombosis 101. Recent studies have shown that 25(OH)D levels are related to mortality from COVID-19. The GC variant of rs2282679 in the gene encoding a vitamin D binding protein is associated with disease severity, and the DHCR7 rs12785878 variant associated with vitamin D deficiency has a higher prevalence in the Portuguese population than in Europe and may explain more severity of COVID-19 in the Portuguese population 102. Some scholars have suggested that using the combination of dexamethasone and vitamin D in COVID-19 may improve the symptom of ARDS 103. Dexamethasone, a corticosteroid, has been identified as an effective immunosuppressant that weakens the innate immune response. Moderate to severe ARDS group treated using dexamethasone has been shown to decrease mechanical ventilation and overall mortality 104. Vitamin D promotes the production of antimicrobial peptides, enhances innate cellular immunity 105, regulates the expression of TLR2 and TLR4, and reduces IL6 levels 106, 107. In addition, vitamin D is an effective renin inhibitor, and a vitamin D deficiency leads to increased AGT expression and the activation of RAAS 108, which reduces the risk of ARDS, myocarditis, or heart injury in patients with COVID-19 109. Vitamin D not only enhances host immunity to avoid viral infection but also inhibits severe cytokine storm and RAAS imbalance, suggesting that it can be an important cryptic adjuvant drug for safely and effectively preventing and curing COVID-19. A new study showed that the hyperlipidaemia drug fenofibrate reduced the stability of the SARS-CoV-2 S1 protein, reducing infection by 70% 110. In addition, fenofibrate can inhibit inflammation and reduce fibrinogen levels and cytokine production, including TNF-α, IL6, and IL1-β 111, 112. The antithrombotic activity of fenofibrate may reduce or prevent adverse events, with good safety and low cost, therefore, we strongly recommend clinical trials for fenofibrate treating COVID-19 patients requiring hospitalization.

Ligands are used as immunogenic adjuvants to activate TLR and NLR for enhancing the efficacy of vaccines and promoting a strong immune response 113. Early activating immune regulation and the innate immune system to enhance antiviral ability may be important ways to prevent COVID-19. After the initiation of inflammatory bodies, NLRP3 affects cellular pressure caused by various pathogens 114. QS-21, aluminium hydroxide, and other immune adjuvants can activate NLRP3 115, 116. Early TLR activation can improve antiviral immunity, and the TLR4 agonist MPL is an adjuvant used in vaccines to prevent HPV and HBV infection 117. Various epitope-based peptide and recombinant S1 subunit vaccine components can activate TLR to protect against SARS-CoV-2 118, 119; however, clinical trials are lacking. A mixture of TLR4 and TLR9 agonists prepared as a vaccine was more effective than a vaccine targeting TLR4 120. The synergistic effects of different receptor agonists for NLPR3 and TLR can activate vaccine adjuvants, promote an innate immune response, and provide solid and lasting protection against pathogens. This may be an important method for the clinical application of vaccines to prevent infection. However, excessive activation of TLR and NLR may lead to the development of cytokine storms, leading to severe COVID-19 121. The role of TLR and NLR during SARS-CoV-2 and their harmful effects need to be determined in further studies.

In this study, the genes associated with both three common comorbidities and COVID-19 were screened through various databases. Candidate genes for the prediction of susceptibility and severity of COVID-19 included *TLR4, NLRP3, MBL2, IL6, IL1RN, IL1B, CX3CR1, CCR5, AGT, ACE,* and* F2*. Studies of COVID-19 have evaluated the roles of polymorphisms in *ACE2, TMPRSS2, IL-6, ACE, MBL2, AGT*, and *CCR5*. However, the roles of polymorphisms in* F2, NLRP3, TLR4, CX3CR, IL1RN,* and* IL1B* in COVID-19 have not been established. This study could provide a reference for subsequent studies of susceptibility genes and marker genes of COVID-19 severity, and suggest that there are shared genetic mechanisms underlying COVID-19 and comorbidities, and reveal potential treatments. These results are of particular value for screening high-risk groups and for improving treatment efficacy and the development of personalised treatments, especially for COVID-19 individuals with the three comorbidities.

However, this study had some limitations. Database-based genomic data only provided a preliminarily explanation of the connection between three common comorbidities and COVID-19. The results should be validated in large population studies as well as functional assays. Additionally, this study focused on three common comorbidities. Other potential factors, such as smoking, obesity, and region, were not included in this study, and more mechanisms underlying associations between multiple factors and COVID-19 need to be explored. Basic and clinical research on related genes and drugs is required to provide more strategies for preventing and curing COVID-19.

## Supplementary Material

Supplementary file.

Supplementary table 1.

Supplementary table 2.

Supplementary table 3.

## Figures and Tables

**Table 1:** Enriched GO-terms of intersection genes

ID	Description	Gene	Count	p	q
GO:0071222	cellular response to lipopolysaccharide	TLR4/NLRP3/IL6/IL1B/CX3CR1/CCR5	6	4.48E-07	1.32E-07
GO:0051091	positive regulation of DNA-binding transcription factor activity	TLR4/NLRP3/IL6/IL1B/CX3CR1/AGT	6	7.63E-07	2.26E-07
GO:0071219	cellular response to molecule of bacterial origin	TLR4/NLRP3/IL6/IL1B/CX3CR1/CCR5	6	4.48E-07	1.32E-07
GO:0032496	response to lipopolysaccharide	TLR4/NLRP3/IL6/IL1B/CX3CR1/CCR5	6	1.84E-06	5.44E-07
GO:0071216	cellular response to biotic stimulus	TLR4/NLRP3/IL6/IL1B/CX3CR1/CCR5	6	5.74E-07	1.70E-07
GO:0009897	external side of plasma membrane	TLR4/CX3CR1/CCR5/ACE/F2	5	2.26E-05	9.52E-06
GO:0048018	receptor ligand activity	IL6/IL1RN/IL1B/AGT/F2	5	0.000196	6.96E-05
GO:0030546	signaling receptor activator activity	IL6/IL1RN/IL1B/AGT/F2	5	0.000196	6.96E-05
GO:0008083	growth factor activity	IL6/AGT/F2	3	0.000753	0.000268
GO:0019955	cytokine binding	IL1RN/CX3CR1/CCR5	3	0.000753	0.000268
GO:0070851	growth factor receptor binding	IL6/IL1RN/IL1B	3	0.000753	0.000268

Notes: Count value represents the number of target genes that core genes belong to in the term, and p was corrected by Benjamini-Hochberg method. And q value was estimated for false discovery rate control. The values p < 0.01 and q < 0.01 were considered statistically significant.

**Table 2:** Enriched KEGG pathways of intersection genes

ID	Description	Gene	Count	p	q
hsa05171	Coronavirus disease -COVID-19	TLR4/NLRP3/MBL2/IL6/IL1B/ACE/F2	7	3.52E-07	1.49E-07
hsa04060	Cytokine-cytokine receptor interaction	IL6/IL1RN/IL1B/CX3CR1/CCR5	5	0.000325	0.000138
hsa05130	Pathogenic Escherichia coli infection	TLR4/NLRP3/IL6/IL1B/F2	5	9.03E-05	0.000038
hsa05133	Pertussis	TLR4/NLRP3/IL6/IL1B	4	9.03E-05	0.000038
hsa05142	Chagas disease	TLR4/IL6/IL1B/ACE	4	0.000149	0.000063
hsa05135	Yersinia infection	TLR4/NLRP3/IL6/IL1B	4	0.000325	0.000138
hsa05164	Influenza A	TLR4/NLRP3/IL6/IL1B	4	0.000523	0.000222
hsa04621	NOD-like receptor signaling pathway	TLR4/NLRP3/IL6/IL1B	4	0.000578	0.000245
hsa05417	Lipid and atherosclerosis	TLR4/NLRP3/IL6/IL1B	4	0.000937	0.000397
hsa05132	Salmonella infection	TLR4/NLRP3/IL6/IL1B	4	0.001287	0.000545
hsa05144	Malaria	TLR4/IL6/IL1B	3	0.000412	0.000175
hsa05134	Legionellosis	TLR4/IL6/IL1B	3	0.000523	0.000222
hsa05321	Inflammatory bowel disease	TLR4/IL6/IL1B	3	0.000578	0.000245
hsa05410	Hypertrophic cardiomyopathy	IL6/AGT/ACE	3	0.001287	0.000545
hsa05323	Rheumatoid arthritis	TLR4/IL6/IL1B	3	0.001287	0.000545
hsa04061	Viral protein interaction with cytokine and cytokine receptor	IL6/CX3CR1/CCR5	3	0.001287	0.000545
hsa04933	AGE-RAGE signaling pathway in diabetic complications	IL6/IL1B/AGT	3	0.001287	0.000545
hsa05146	Amoebiasis	TLR4/IL6/IL1B	3	0.001287	0.000545
hsa04620	Toll-like receptor signaling pathway	TLR4/IL6/IL1B	3	0.001287	0.000545
hsa04625	C-type lectin receptor signaling pathway	NLRP3/IL6/IL1B	3	0.001287	0.000545
hsa05162	Measles	TLR4/IL6/IL1B	3	0.002715	0.001150
hsa04217	Necroptosis	TLR4/NLRP3/IL1B	3	0.003568	0.001511
hsa05152	Tuberculosis	TLR4/IL6/IL1B	3	0.004729	0.002003
hsa05163	Human cytomegalovirus infection	IL6/IL1B/CCR5	3	0.00864	0.003660
hsa04614	Renin-angiotensin system	AGT/ACE	2	0.001628	0.000690
hsa01523	Antifolate resistance	IL6/IL1B	2	0.002715	0.001150
hsa05143	African trypanosomiasis	IL6/IL1B	2	0.003568	0.001511
hsa05332	Graft-versus-host disease	IL6/IL1B	2	0.004412	0.001869
hsa04623	Cytosolic DNA-sensing pathway	IL6/IL1B	2	0.008833	0.003742

Notes: Count value represents the number of target genes that core genes belong to in the term, and p was corrected by Benjamini-Hochberg method. And q value was estimated for false discovery rate control. The values p < 0.01 and q < 0.01 were considered statistically significant.

## Abbreviations

GO: Gene Ontology
KEGG: Kyoto Encyclopedia of Genes and Genomes
NLRP: NOD-like receptor protein
PPI: protein-protein interaction
TMPRSS2: transmembrane serine protease 2
TLR: Toll-like receptor
RBD: receptor-binding domain
ROS: reactive oxygen species
CRAC: Ca^2+^ release-activated Ca^2+^ channels
RAAS: renin-angiotensin-aldosterone system
ACE: angiotensin-converting enzyme
ARDS: acute respiratory distress syndrome
